# Pregnancy and Dentistry: A Literature Review on Risk Management during Dental Surgical Procedures

**DOI:** 10.3390/dj9040046

**Published:** 2021-04-19

**Authors:** Vittorio Favero, Christian Bacci, Andrea Volpato, Michela Bandiera, Lorenzo Favero, Gastone Zanette

**Affiliations:** 1 Unit of Maxillofacial Surgery and Dentistry, University of Verona, 37129 Verona, Italy; vittorio.favero@univr.it; 2 Dental Clinic, Department of Neuroscience, University of Padua, 35129 Padua, Italy; christian.bacci@unipd.it (C.B.); michela.bandiera@unipd.it (M.B.); lorenzo.favero@unipd.it (L.F.); gastone.zanette@unipd.it (G.Z.)

**Keywords:** pregnancy, dentistry, oral surgery, risk management

## Abstract

**Background:** Pregnancy is a unique moment in a woman’s life, accompanied with several physiologic changes that have an impact on oral health. **Aim of the study:** The purpose of the present study was to conduct a critical review of published literature regarding pregnancy and dentistry, the most frequent oral diseases that are encountered during pregnancy, their correlation to adverse pregnancy events, and safe dental treatments that can be performed during pregnancy. **Methods:** A Medline/COCHRANE search was carried using specific keywords and MeSH terms, combined with the boolean operators “OR” and “AND”. **Results:** The search led to 146 publications including guidelines, meta-analyses, systematic and non-systematic reviews, published between 2000 and 2021. **Discussion and conclusions:** Due to the increased inflammatory and immune body response that characterizes pregnancy, periodontal conditions are often aggravated during pregnancy and periodontal disease encountered frequently in pregnant patients. There are conflicting study results in the literature regarding the association between periodontitis and adverse pregnancy outcomes. Periodontal treatment did not show a significant reduction in the adverse outcomes. Many dentists, often due to lack of information, are reluctant to provide dental treatment to pregnant women. However, preventive and restorative dental treatment is safe during pregnancy. Diagnostic radiographs may be performed after the first trimester if absolutely necessary. Analgesics (such as paracetamol) and anesthetics (such as lidocaine) are also considered safe. In case of infection, antibacterial drugs such as amoxicillin, ampicillin, and some cephalosporines and macrolides can also be prescribed. Organogenesis takes place in the first trimester, the time during which the fetus is susceptible to severe malformations (teratogenesis). The ideal time to perform dental treatment is the second trimester (week 17 to 28). However, acute pain or infections make the intervention of the dentist absolutely necessary and emergency treatment can be performed during the whole pregnancy period.

## 1. Introduction

The pregnant woman undergoes numerous physiological changes. The changes can be systemic and local, such as those that occur in the oral cavity. Since oral health is an integral part of general health, the problems in the oral cavity encountered in pregnant women must be addressed promptly. It is very important that the dentist takes into account the physiological changes that occur throughout pregnancy and be aware that his intervention through dental treatments may have effects on the lives of two people (the mother and the baby). Consequently, the clinician should adopt all measures necessary to minimize the risk of adverse events [1].

The changes that occur during pregnancy involve the cardiovascular, respiratory, renal, gastrointestinal, and hematological systems.

Plasma volume and blood cell mass increase to provide the increased amount of oxygen required by maternal and placental tissues. The heart rate increases to meet the fetus’ needs. From the second trimester there is a decrease in blood pressure that can lead to hypotensive supine syndrome [2]. The changes involving the plasma levels of the coagulation factors VII, VIII, IX, X, and XI, fibrinogen increase, and the increase in the number of leukocytes and erythrocytes put pregnant women at higher risk for developing thromboembolic phenomena, such as deep vein thrombosis and pulmonary embolism [3,4].

Breathing becomes more difficult due to the push of the uterus into the diaphragm. Lung residual functional capacity decreases up to 20% and dyspnea episodes become more frequent during the third trimester (up to 75% of pregnant women) [5].

Pregnant women experience predilection or aversion towards certain types of food. Gastric and intestinal emptying slows down in order to maximize the absorption of nutrients causing digestive difficulties, nausea, vomiting, constipation, heartburn, etc. [6]. Increased acidity in the oral cavity due to vomiting can lead to enamel erosion (perimolysis) [7]. The dentist should investigate the frequency and duration of nausea and/or vomiting.

Glomerular filtration rate increases during pregnancy and, as a consequence of this, the clearance of creatinine, uric acid, and urea increases as well. The bladder is pushed up anteriorly due to uterine compression. Some of the most frequent complaints of pregnant women are the increased frequency of urination and the increased risk of contracting urinary tract infections. The dosage of renal excretion drugs prescribed during pregnancy should be increased due to their faster clearance [8].

Pregnant women are also at risk of developing gestational diabetes, due to the endocrine system changes that occur during pregnancy. This risk is higher in obese women with a family history of type II diabetes mellitus [9].

### 1.1. Orofacial District

The physiological changes that occur in the mouth during pregnancy are widely documented in the literature. The lack of routine dental check-ups and the postponement of dental treatments expose the pregnant patient to a greater risk of dental infections. The immunological changes during pregnancy influence oral health. In particular, the suppression of some neutrophil functions may explain the aggravation of gingivitis. Neutrophil function suppression is a relevant factor in the association between periodontal disease and pregnancy [10,11].

Increased capillary permeability due to high levels of estrogen in the blood predispose pregnant women to gingivitis and hyperplasia [12]. 

### 1.2. Pharmacology during Pregnancy

The distribution, degradation, and elimination of drugs undergoes some changes during pregnancy. The pharmacokinetics of drugs changes and, therefore, under certain circumstances, their effect (pharmacodynamics) as well. 

The decreased production of hydrochloric acid by the gastrointestinal system has consequences on drug absorption and bioavailability. Changes in liver enzyme production, on the other hand, can alter the activation of prodrugs, the process of absorption, metabolism, and offset. Codeine, which is converted into morphine by the cytochrome CYP2D6, whose activity is increased during pregnancy, can be an example of this phenomenon. Codeine brings rapid pain relief in pregnant women, but it is also accompanied by higher toxicity [13,14]. In pregnant patients, body mass and volume increase and, therefore, there is a greater drug distribution volume and clearance [15]. Since both clearance and distribution volume increase during pregnancy, changes in the half-life of drugs cannot be predicted, and each drug should be studied individually [16].

The placenta is a semipermeable barrier to the passage of substances, similar to the blood–brain barrier. In general, all drugs that can cross the blood–brain barrier can also cross the placenta. 

### 1.3. Adverse Events during Pregnancy

The sporadic spontaneous abortion occurs in the first trimester, with an incidence of 10–15% in all clinically recognized pregnancies [17]. The etiology of spontaneous abortions includes endocrine factors and uterine malformations, but most cases are due to chromosomal anomalies.

Preterm birth, or premature birth, is the birth of a baby before the completion of 37 weeks of gestation [18]. Etiological factors include infection, increased uterine volume, iatrogenic causes, and idiopathic factors.

Preeclampsia is a pregnancy complication characterized by high blood pressure (at least 140/90 mmHg), proteinuria, and edema [19]. It occurs in 3–7% of pregnant women.

The World Health Organization (WHO) recommends calcium supplementation during pregnancy for women that have low calcium intake through diet [20]. Studies have shown that calcium supplementation during pregnancy may reduce the risk of preeclampsia. It also helps prevent preterm birth [21]. 

Gestational diabetes mellitus affects 3–7% of pregnant women and its incidence is rising [22,23]. Pregnant women who develop gestational diabetes mellitus are at higher risk of developing periodontal disease. Moreover, once the periodontal disease has developed, it makes diabetes control more difficult. The presence of untreated periodontal disease determines a state of general inflammation which contributes to the increase of blood glucose levels. Therefore, it is very important that clinical signs of periodontitis in diabetic patients are intercepted at an early stage [24].

Organogenesis (the development of organs) takes place in the first ten weeks of embryonic life. Consequently, to consider an environmental factor as teratogenic or capable of producing anomalies in the embryo, exposure to it must occur during this particular period [25]. 

## 2. Aim

The present study aims to carry out a review of the international scientific literature concerning Dentistry and Pregnancy in order to evaluate:

− the most frequent oral diseases during pregnancy, their causes and risk factors;
− the correlation of oral pathology to adverse events concerning the pregnant woman and the newborn;
− the diagnostic procedures, dental and pharmacological treatments that can be performed safely during pregnancy, including contraindications;
− precautions that should be taken by the dentist during oral procedures.

## 3. Methods

The research was carried out by identifying the Medical Subject Headings (MeSH) specific to this topic, in the thesaurus dictionary controlled by the National Library of Medicine (NLM), and non-MeSH words, using them to search for the work—individually or in association with each other—through the use of the boolean operators “AND”, “OR”: Pregnancy; Pregnant Patient; Pregnant Women; Dentistry; Dental Care; Dental Treatment; Oral Health; Oral Hygiene; Periodontitis; Periodontal Disease.

The articles were selected based on the following criteria:

Publication dates: from 1 January 2000 to 2021; Languages: English, Italian; Article types: Guidelines, Meta-analysis, Systematic Reviews, Reviews.

The articles must report the relationship between pregnancy and oral health, dentists’ knowledge of pregnancy and its effects on oral health, the possible biological correlation between diseases of the oral cavity and the incidence of perinatal adverse events and, finally, the additional precautions that the dentist must implement in the diagnosis and treatment of the pregnant woman. The search results were screened by reading the titles and abstracts. All articles that were not related to the scope of this review were excluded. Studies not including human subjects were excluded.

## 4. Results

The search led to 373 results. Of each article, abstracts and titles were examined. Articles that were not related to the topic were excluded. We then obtained the full texts of all potentially eligible articles. After the complete reading, 146 articles were selected, discarding the remaining ones, deemed unsuitable because they were not pertinent to the purpose of the study. Titles that were repeated in the various searches were also excluded. Articles published after 1 January 2000 concerning the following topics were selected: knowledge of the dentist regarding pregnancy and its effects, the possible correlation between diseases of the oral cavity (especially periodontal disease) and the incidence of perinatal adverse events, and, finally, the additional precautions that the clinician must implement in the diagnosis and therapy of the pregnant woman.

A further selection was made based on the level of scientific evidence, taking into greater consideration 46 publications including systematic guidelines, meta-analysis, and reviews, extending the search to non-systematic reviews if the results were not satisfactory.

The remaining 100 articles were not altogether excluded from this work but were deemed to be less linked to the subject of the review and corresponding to a lower level of scientific evidence.

## 5. Discussion

### 5.1. Oral Pathology during Pregnancy

#### 5.1.1. Periodontal Disease

Gingivitis induced by plaque accumulation is the most frequent condition encountered during pregnancy (60–75% of pregnant women) [26], hence the importance of establishing rules for prevention and periodontal treatment. Gingivitis usually appears between the third and eighth month of gestation, and gradually diminishes after childbirth. While gingivitis usually occurs as a result of poor oral hygiene and/or local inflammation, mediated by plaque bacteria, the changes that accompany pregnancy often increase the body’s response to local inflammatory agents: gingivitis is characterized by dark red swollen gingiva that easily bleeds and which is, in fact, a sign of altered vascularization [27].

A recent study reported the association between the worsening of periodontal disease and increased “red complex” bacteria such as *Porphyromonas gingivalis* and *Prevotella intermedia*. However, the proportions between bacteria do not change during pregnancy [28]. Another study measured the bacterial load in pregnant and non-pregnant women and found that the level of *Campylobacter rectus* was higher in pregnant women. This result is explained by the fact that the level of this bacteria is directly related to the estradiol levels in the body [29]. In another study, on the contrary, the bacteria, in particular, the *Fusobacterium nucleatum* which can cross the placental barrier and cause acute infections, showed no variation in pregnant and non-pregnant women [30].

In conclusion, the major cause of periodontal disease exacerbation in pregnant women is not to be found in plaque, whose pathogenic quantitative and qualitative composition is not varied. During pregnancy, the inflammatory response is highly activated [31], as witnessed by the increased expression of inflammatory markers [32].

Periodontal disease treatment includes non-surgical and surgical treatments, performed alone or combined. The most common periodontal therapy is motivating the patient and giving instructions on how to maintain good oral hygiene, how to prevent plaque build-up and tartar formation [33,34]. Recent publications report that behavioral and educational interventions during pregnancy can improve periodontal health. These interventions are more effective if carried out not only at the beginning of pregnancy but throughout the whole pregnancy period [34,35].

#### 5.1.2. Tooth Mobility

Generalized tooth mobility in pregnant women is related to the degree of involvement of the periodontium and the changes in the mineralization of the hard lamina [36]. Longitudinal studies have shown that the probing depth increases because of swollen gums and inflammation [37]. While most studies conclude that the clinical attachment loss (CAL) is temporary [38,39], others say it does not regress at the end of pregnancy [40].

#### 5.1.3. Decay

Changes in salivary composition during pregnancy and breastfeeding can temporarily predispose teeth to erosion and decay [41]. There are, however, no studies that have shown increased incidence of decay during pregnancy or in the immediate postpartum period. In any case, pre-existing and untreated caries have a greater risk of progressing. 

#### 5.1.4. Erosion

Nausea and vomiting are very common in pregnant women: 70–85% of women experience these symptoms, which tend to disappear after the first trimester. Although nausea and vomiting are present during the early stages of pregnancy, some women (0.3–2%) do not have a remission of symptoms. Moreover, some women may suffer from hyperemesis gravidarum, a severe form of nausea and vomiting that can lead to enamel loss, especially in their vestibular surface, due to acid-induced erosion [7,42].

#### 5.1.5. Epulis Gravidarum

Epulis gravidarum is also referred to as granuloma or pregnancy tumor. The lesion is similar to a benign tumor caused by hyperplasia of the gingival connective tissue. Epulis gravidarum is most frequently encountered in areas of gingival inflammation or chronic trauma [43]. Plaque and tartar deposits are often found in areas adjacent to the lesion. Subgingival scaling and root planing, together with patient education and oral hygiene instructions, should be performed before delivery to reduce plaque retention as much as possible [26]. Generally, the epulis gravidarum spontaneously regresses after childbirth, although surgical excision is necessary in some cases. It is essential to warn the patient that the need for surgical excision may occur before the end of pregnancy [44].

### 5.2. Periodontal Disease and Adverse Events during Pregnancy

Adverse events during pregnancy are painful experiences in women’s lives and also have great costs due to their implications. The World Health Organization has reported that preterm birth is one of the main causes of death for children under 5 years of age [45]. Some observational studies have encouraged periodontal treatment during pregnancy as an attempt to reduce the risk of premature birth and other adverse effects. 

### 5.3. Preterm Birth

Preterm birth is the adverse event of pregnancy most frequently associated with periodontal disease. Low birth weight and preeclampsia are also associated with periodontal disease [46]. Periodontitis is known to have effects on cardiovascular disease, diabetes, chronic lung disease, and also pregnancy outcomes [47]. Since periodontal disease is characterized by a medium-low degree of inflammation, it has been hypothesized that patients with periodontal disease have a higher risk of ending the pregnancy negatively. Several publications have highlighted the positive association between periodontal disease and adverse pregnancy outcomes [48,49,50,51,52,53,54]. These studies, however, do not use the same clinical measurement method, thus leading to conflicting results. Consequently, the role of periodontal disease in adverse pregnancy outcomes remains an unresolved issue. The only proven fact is that periodontal disease is likely to worsen during pregnancy [32]. Some observational studies on humans have reported a positive association between periodontal disease and adverse outcomes of pregnancy, including preterm birth [55,56,57], premature rupture of membranes [58], preeclampsia [59], abortion [60], and postpartum endometritis.

However, not all observational studies report an association between preterm birth or low birth weight and periodontal disease: Davenport et al. published a case–control study in 2002, in which 236 premature and 507 term infants were enrolled. They reported lower risk of preterm and low birth weight in patients with increased pocket depth measured during labor. The authors, therefore, concluded that their results did not support the hypothesis that improving periodontal health of pregnant women leads to better pregnancy outcomes [61]. Moore et al., in a large cohort study and a smaller case–control study, did not find any correlation between preterm birth and periodontal disease [62,63]. Lafaurie et al., in a case–control study, concluded that the presence of periodontal pockets was not a risk factor for preeclampsia, but they reported an association between periodontal pockets and premature rupture of membranes, low birth weight, and preterm birth [64].

#### 5.3.1. Periodontal Disease and Its Correlation with Preeclampsia

Preeclampsia is characterized by two syndromes, both resulting in high mortality and morbidity rates [65]: on the one hand, there is the maternal syndrome, characterized by activation of the endothelium cells, disturbances in blood volume, and control of blood pressure, proteinuria, and edema. On the other, there is fetal syndrome, mainly represented by the reduction in intrauterine growth [65,66,67,68]. Several studies conducted in the past have suggested the existence of an association between periodontal disease and increased risk of developing preeclampsia [69,70,71,72,73], but the results have been controversial. In 2013, Sgolastra et al. published a meta-analysis that ascertained the scientific evidence on the possible association between periodontal disease and preeclampsia: a positive correlation between the two pathologies emerged from the analysis of 15 published studies, but due to the significant heterogeneity of the studies in the definition and diagnosis of periodontal disease as well as the poor methodological quality, the meta-analysis indicates the need for subsequent studies [65].

In the same year, Wei et al. advanced the hypothesis that the association may reflect the induction of periodontal disease from the preeclamptic condition, or that it may instead be a reflection of the increased inflammatory response during pregnancy [50]. The following year, however, Huang et al. denied the possibility that periodontal treatment might have positive effects in reducing the risk of preeclampsia [74].

#### 5.3.2. Role of Periodontal Disease Treatment

The mechanism of action of periodontal therapy in preventing the adverse events of pregnancy is not yet clear. Generally, the main purpose of periodontal treatment is to resolve inflammation by minimizing plaque and tartar levels in order to prevent (or limit) the destruction of tissues, preserve the teeth and aesthetics, and minimize discomfort [75].

In 2003, a systematic review of nine observational and three interventional studies advanced the hypothesis that there was some evidence of the possibility that periodontal treatment could reduce the adverse outcomes of pregnancy [76].

The COCHRANE review of Iheozofor-Ejiofor et al., published in 2017, compared fifteen randomized clinical trials that recruited pregnant women with periodontitis or gingivitis in order to evaluate how periodontal treatment influenced preterm delivery. In the eleven studies comparing women undergoing treatment and women not undergoing any treatment, the meta-analysis did not show a significant difference in preterm birth (less than 37 weeks of gestation). However, there is low-quality scientific evidence that the treatment can reduce the risk of low birth weight (babies weighing less than 2500 g). The other four studies confronted traditional periodontal treatment to alternative treatments, but it was not possible to scientifically compare the data because of the heterogeneity of the studies [77].

In conclusion, it is not clear whether treating the periodontal disease during pregnancy could have an impact on preterm birth. There is low-quality scientific evidence that it can reduce the risk of low birth weight (<2500 g) and there are insufficient data to determine which periodontal treatment is better in preventing adverse outcomes [77]. Da Silva et al., in a recent meta-analysis, showed that periodontal treatment during pregnancy decreased the levels of inflammatory biomarkers in the crevicular fluid (interleukins, chemokines, prostaglandins, TNF), but there were no significant results on the blood levels of the umbilical cord; the incidence of adverse events had not decreased [78].

### 5.4. Dental Diagnosis in the Pregnant Woman

#### 5.4.1. Dentists’ Knowledge Regarding Pregnancy

Despite the existing scientific evidence of changes and modifications that occur in the body during pregnancy, many dentists are reluctant to provide dental care to pregnant women. This is due to a lack of preparation, leading to repercussions and aggravations of the oral problem, with consequences that can cause harm to both the mother and the child. A literature review published in 2013 by Pontes Vieira et al. collected data from 4184 dentists, who were questioned about the management of pregnant women. What emerged was that a high percentage of dentists did not have sufficient information on how to treat pregnant women. In one of the analyzed studies [79], 40% of the participants would never perform an X-ray on a pregnant woman. Uncertainty persists even when considering the use of anesthetics and analgesics: in one of the studies, three-quarters of the participants were reluctant to provide drugs that would reduce pain and swelling in an acute dental emergency situation [80].

Around 46% of the dentists that participated in the study conducted by Navarro et al. did not recommend the use of vasoconstrictor in association with analgesic. Regarding the time in which it is preferable to perform dental treatments, it is known that the best time is the second trimester. However, some dentists, out of fear or ignorance, advise not performing any treatment at all during pregnancy [80,81,82].

#### 5.4.2. Medical History (Anamnesis)

Medical history is a fundamental part of the dental examination. Its importance is even greater in patients who are in particular pathophysiological conditions, such as pregnancy. Even though the clinician must always assume that the woman of childbearing potential might be pregnant, it is good that he explicitly asks the patient about it. The clinician should collect a detailed medical and dental history through a specific questionnaire or an interview. The dentist must also investigate eating habits, including the use of tobacco, alcohol, or recreational drugs. The clinician should also reassure the patient in every way, explaining that dental treatment, as well as the use of radiographs, analgesics, and local anesthetics, are safe even during pregnancy [83].

Informed consent is at the base of medical ethics and forensic medicine; the patient should be provided with all the information regarding the risks, benefits, and treatment alternatives available to meet her needs. It is always recommended to receive written informed consent from the patient for all surgical or invasive procedures. However, specific consent for pregnancy is not required [44].

#### 5.4.3. Dental Physical Examination

The dental examination must be performed accurately, with a particular focus on carious lesions and signs of periodontal disease [83].

#### 5.4.4. Instrumental Diagnosis

Radiographs are crucial for diagnosing and treating dental problems. They are considered safe even during pregnancy [84,85]. The patient should be exposed to very low doses of radiation, to minimize the risk of potentially harmful effects. The Food and Drug Administration (FDA) has published recommendations on radiographs, formulated by a group of dental health experts [86], and their recommendations remain valid even during pregnancy: the operator should not subject the patient to more X-rays than necessary; he should provide the patient with protection against exposure to the woman’s abdomen and neck and use the technique of long cones with the appropriate centering devices. In article 10, the Legislative Decree 187/00 of the Italian Ministry of Health expresses itself regarding the health protection of pregnant women against ionizing radiation: the operator is required to inquire if the woman is pregnant and to consider the real need and urgency of the radiography, especially if the radiation dose that will reach the uterus is higher than 1 mSv [87].

Digital radiography is also safe during pregnancy. It offers the advantage of reduced radiation, of not needing films and chemical processes, and provides the images practically instantly, making it possible to print them later [44]. However, dentists should consider the gestational age of the fetus. During the first trimester, when organogenesis takes place, the fetus is more susceptible to radiation. Doses that are considered safe during the second and third trimesters may be harmful during the first trimester. Nevertheless, high doses of radiation (greater than 0.5 Gy or 50 rad) should be avoided throughout pregnancy [88].

### 5.5. Dental Treatment in Pregnant Women

Dental emergencies, acute pain, and infections make the dentist’s intervention necessary, and treatment should not be postponed.

The American Academy of Periodontology advises dental professionals to treat acute periodontal infections or infectious foci regardless of the pregnancy stage [89]. Caries treatment is recommended to reduce the level of bacteria causing the disease. If the pregnant woman does not undergo conservative treatment, her baby’s chances of acquiring cariogenic bacteria increase due to the transmission through saliva from the mother to the child [90]. 

Research shows that while nonsurgical periodontal therapy during the second trimester is safe, it does not reduce the incidence of negative pregnancy outcomes [91]. The main role of periodontal treatment during pregnancy is to improve the periodontal and general health of the pregnant woman. Non-surgical periodontal therapy improves the periodontal conditions of most pregnant women with periodontal disease. The use of local anesthesia is also considered safe [92]. Although said studies were performed during 13–23 weeks of gestation, it does not mean that the treatment performed before or after is not equally safe.

It is fundamental to remember that emotions and anxiety are accentuated during pregnancy, and this can intensify the fear and perception of pain in the dental chair [93].

#### 5.5.1. The Positioning of the Patient

Pregnant women, during the third trimester, are at risk of experiencing hypotensive supine syndrome. When the patient is lying in supine position, the enlarged uterus presses against the vena cava, which carries blood to the heart. This pressure exerted by the fetus causes a sudden drop in blood pressure. To avoid dizziness and fainting, the dentist should position the patient in semi-reclined position. A maneuver that could help is to tell the patient to move to the left side or place a cushion under the right-side lower back, to move the uterus towards the aorta, which does not collapse so easily [44].

#### 5.5.2. Drugs during Pregnancy

The potential risk of drugs in causing birth defects if used during pregnancy is expressed through the classification of the Food and Drug Administration (FDA). The FDA has divided drugs into five categories based on the reliability of existing scientific evidence and the cost/benefit ratio [94].

Category A: Adequate and well-controlled studies have failed to demonstrate a risk to the fetus in the first trimester of pregnancy (and there is no evidence of risk in later trimesters).
Category B: Animal reproduction studies have failed to demonstrate a risk to the fetus and there are no adequate and well-controlled studies in pregnant women.
Category C: Animal reproduction studies have shown an adverse effect on the fetus and there are no adequate and well-controlled studies in humans, but potential benefits may warrant use of the drug in pregnant women despite potential risks.
Category D: There is positive evidence of human fetal risk based on adverse reaction data from investigational or marketing experience or studies in humans, but potential benefits may warrant use of the drug in pregnant women despite potential risks.
Category X: Studies in animals or humans have demonstrated fetal abnormalities and/or there is positive evidence of human fetal risk based on adverse reaction data from investigational or marketing experience, and the risks involved in use of the drug in pregnant women clearly outweigh potential benefits [94].

#### 5.5.3. Local Anesthetics

All local anesthetics can cross the placental barrier, with the possibility of producing effects on the fetus. These drugs can cause cardiovascular and neurological toxicity. Lidocaine is the most used local anesthetic during pregnancy; the proportion of lidocaine that circulates freely, i.e., not linked to transport proteins, is high and, therefore, the quantity of lidocaine transferred from the mother to the fetus is also high. Vasoconstrictors, such as epinephrine, are often added to lidocaine to increase the duration of its effect and to reduce toxicity. Epinephrine-induced vasoconstriction delays the absorption of the anesthetic and, therefore, the level of lidocaine in the blood increases gradually and without peaks. The anesthetic is transferred to the fetus just as slowly, with a wider safety margin. Considering that the anesthetic has negligible effects on the fetus even at submaximal doses, the use of lidocaine 2% with epinephrine 1: 100,000 is considered relatively safe [95].

Some publications have documented fetal bradycardia due to the use of bupivacaine or mepivacaine and, therefore, they are classified into category C, as well as articaine, of which there are no controlled studies in pregnant women [96]. In a chair session, a pregnant woman can receive up to 5 tubes containing epinephrine with a concentration of 1:100,000 (or 10 tubes of anesthetic with 1:200,000 concentration of epinephrine) [25].

#### 5.5.4. Antibiotics, Antifungals, and Antiseptics

Antibacterial drugs can be prescribed during pregnancy in the case of ongoing infectious pathological processes, choosing active ingredients with broad safety indices. Beta-lactams such as ampicillin, amoxicillin (not in association with clavulanic acid), and some cephalosporins, and macrolides such as clarithromycin and erythromycin, are considered safe at therapeutic dosages [97]. Instead, tetracyclines such as doxycycline and minocycline, which can cause liver damage to the pregnant woman and dyschromia of the dental enamel of the baby, as well as gentamicin, which causes fetal ototoxicity, should be avoided. As for antifungals, nystatin and clotrimazole are safe, while it is preferable to avoid fluconazole and ketoconazole which are toxic to the fetus [98].

Chlorhexidine (in concentrations 0.05–0.2%) is an antiseptic active ingredient present in many types of mouthwash. It belongs to the FDA category B since animal studies have not shown teratogenicity at high doses, but there are no controlled data obtained from human pregnancies and, therefore, its use in pregnancy is recommended only in case of need [99]. In addition, all products that contain alcohol should be avoided during pregnancy [99].

#### 5.5.5. Analgesics

Acetylsalicylic acid is not recommended due to the risk of postpartum hemorrhage. It is preferable to administer paracetamol, which also causes less gastric inflammation. The use of NSAIDs in the early months of pregnancy is also to be avoided as some authors report increased risk of septal heart defects in the newborns of mothers that have assumed NSAIDs such as ibuprofen, naproxen, and ketoprofen [100]. The new category of cyclooxygenase inhibitors type 2 (celecoxib and rofecoxib) has been classified into category C. These drugs should also be avoided in the first trimester because they could cause premature closure of the arterial duct [1].

#### 5.5.6. Nitrous Oxide

Nitrogen oxide, commonly known as nitrous oxide, is widely used to provide sedation and analgesia during labor. In obstetrics, it is used because it is easily administered, presents minimal toxicity, does not interfere with uterine contractions, and does not cause malignant hyperthermia (a severe reaction caused by exposure to certain general anesthetics). Moreover, in dentistry, the combination of nitrous oxide and oxygen is frequently used. It is safe and ensures excellent sedation for phobic and anxious patients [101,102]. It is not uncommon for pregnant women to be particularly anxious. If the patient’s anxiety levels are as high as to prevent collaboration and iatrosedation is insufficient to manage the state of fear, nitrous oxide is the sedative of choice. Prolonged exposure should be avoided as much as possible.

Appropriate use of aspiration and elimination devices for environmental gases significantly minimize risks [103]. Exposure to protoxide for long periods of time has been associated with decreased fertility, but sedation time during dental treatments is relatively short and does not have deleterious or teratogenic effects [93]. A retrospective study of nearly 6000 pregnant women subjected to general anesthesia, obtained also with the use of nitrous oxide, did not report any adverse effects for neither patient nor fetus [104,105]. It is recommended that nitrous oxide is administered by qualified and trained personnel [44].

#### 5.5.7. Restoration Materials

Amalgam is a restoration material commonly used in the past, now supplanted by composite materials. The mercury present in amalgam fillings is inorganic, unlike the organic mercury found in fish, for example. During chewing, especially chewing gum, and in bruxism, the inorganic mercury of the amalgam restorations is continuously released in the oral cavity during the chewing process (especially chewing gums) and ends up in the bloodstream [106,107]. Similarly, the use of hydrogen peroxide-based toothpastes and whitening products also causes inorganic mercury release from amalgam fillings [108]; therefore, their use is not recommended during pregnancy. The placement and removal of amalgam restorations cause a temporary increase in inorganic mercury levels in the bloodstream [109].

Mercury vapors are inhaled, brought to the lungs where they can enter the bloodstream and reach the fetus through the placenta [110]. The use of the rubber dam and a powerful aspiration system can reduce the amount of inhaled vapors. It is recommended to postpone the removal of amalgam restorations until the end of pregnancy if it is not possible to operate safely. Nevertheless, studies report no adverse effects during amalgam placement or removal [44]. After reviewing 200 studies, FDA reiterated that dental amalgam is a safe and effective material for dental restorations, classifying it as a class II medical device, such as composite materials [111].

Composite resins, glass ionomers, gold, and porcelain are alternative restoration materials to amalgam. Composite resins are made up of polymerized resin and an inorganic filler. Recent studies on methyl methacrylate monomers, such as methyl methacrylate (MMA), hydroxyethyl methacrylate (HEMA), triethylene glycol dimethacrylate (TEGDMA), bisphenol-A (BPA), bisphenol-A glycidyl methacrylate (Bis-GMA), and on bisphenol-A dimethacrylate (Bis-DMA), have shown how the monomers are released into the oral cavity, penetrate the dentin, and may reach the pulp [112,113]. Although BPA may not be a direct component of resin composite or sealants, it may be a byproduct of the degradation of other monomers contained in the restoration materials, caused by salivary enzymes. Joskow et al. reported small traces of BPA in the saliva, a few hours after the dental sealants had been placed. Short-term exposure did not show any health risk, however, there is a lack of data on long-term effects [114].

#### 5.5.8. Surgical Procedures

Under urgent circumstances, dentoalveolar surgery directed towards the elimination of pain, infection, and neoplasia can be performed during pregnancy. These procedures include tooth extractions, incisions, and drainage of dental infections. Clearly, oral surgery during pregnancy should be considered only if absolutely necessary. Surgery for aesthetic purposes such as orthodontic treatment or orthognathic surgery must be delayed to the postpartum period. The second trimester is the safest period to provide dental treatment [115,116,117,118].

Local anesthesia can be administered during oral surgery procedures. The dentist should use anesthetics that are considered safe (such as lidocaine), avoid high doses of epinephrine and be extremely careful during the injection procedure (the intravascular injection of epinephrine causes uterine artery vasoconstriction and decreased blood flow). Radiographs should be performed as little as possible and protective equipment must always be used [6] [119].].

Oral cancers represent nearly 2% of all malignant tumors during pregnancy. The safest period for surgery is the second trimester. The risk of postoperative infection is high because of the physiological suppression of cell-mediated and humoral immunity during pregnancy. Whenever planning to perform oral surgery, the dentist or oral surgeon should consult the patient’s obstetrician before the procedure [120,121,122,123].

## 6. Conclusions

Although the international guidelines clearly express the possibility of performing diagnosis and treatment in pregnant patients, there is still a lot of reluctance from dentists to treat women during pregnancy. This is due to fear, ignorance, and false knowledge. On the other hand, it is often the pregnant woman herself who refuses to undergo any kind of dental treatment. It can be affirmed that although pregnancy is a particular event characterized by many changes that affect also the oral cavity, it is possible and safe to treat pregnant patients who need treatment. There are safe alternatives for each category of drugs that are commonly used in dentistry. The best time to perform the treatment is the second trimester. Published literature states that there is a correlation between pregnancy and exacerbation of gingivitis and periodontitis, but further studies are needed to investigate the association and effects of dental treatment in preventing adverse pregnancy outcomes. In particular, it is necessary to follow a common path in the diagnosis and study parameters of periodontal disease. 

Teratogens are chemical, physical, or biological agents that directly harm the fetus. Some of the abnormalities they cause may be mild and with no relevant repercussions on the fetus, while others are so serious that they might cause death of the fetus or unborn baby. During their development, each apparatus goes through a critical period during which it is particularly susceptible to the action of teratogens. To harm the unborn child, teratogens must act during critical periods of embryological or fetal development, inducing embryopathy or fetopathy. During organogenesis (4–5 to 10 weeks of gestation) fetal tissues begin to differentiate; this is the most susceptible interval to teratogenesis.

We suggest that there should be a direct line between pharmacological research (arrival of new drugs) and adequate communication, not only to obstetricians but to dentists as well. Effort should be made in formulating a joint obstetric–dental document, so that the patient can have in his hand a clear explanation of pharmacology during pregnancy. This is necessary for eliminating doubts, uncertainties, or unnecessary suffering of the mother and the developing baby.
